# *Meloidogyne incognita* - rice (*Oryza sativa*) interaction: a new model system to study plant-root-knot nematode interactions in monocotyledons

**DOI:** 10.1186/s12284-014-0023-4

**Published:** 2014-09-22

**Authors:** Phong Vũ Nguyễn, Stéphane Bellafiore, Anne-Sophie Petitot, Rana Haidar, Aurélie Bak, Amina Abed, Pascal Gantet, Itamara Mezzalira, Janice de Almeida Engler, Diana Fernandez

**Affiliations:** Institut de Recherche pour le Développement, UMR 186 IRD-Cirad-UM2 Résistance des Plantes aux Bioagresseurs, 911 avenue Agropolis, Montpellier, 34394 Cedex 5 France; UMR IBSV INRA/CNRS/UNS, 400, Route de Chappes, Sophia Antipolis, F-06903 CEDEX France; Université Montpellier 2, UMR IRD-UM2 DIADE, 911 avenue Agropolis, Montpellier, 34394 Cedex 5 France; INRAA- CRP, BP 37 Mehdi Boualem, Baraki, Algiers Algeria; Institut de Recherche pour le Développement, LMI RICE, University of Science and Technology of Hanoi, Agricultural Genetics Institute, Hanoi, Vietnam; Embrapa - Recursos Genéticos e Biotecnologia, Brasília, DF 70849-970 Brazil; Nông Lâm University, Linh Trung, Thủ Đức, Hồ Chí Minh city, Việt Nam; INRA, UMR1065 Santé et Agroécologie du Vignoble (SAVE), ISVV, CS, 20032, 33882 Villenave d'Ornon, France

**Keywords:** Rice, Root-knot nematodes, Giant cells, Immunity suppression, Meloidogyne graminicola, Meloidogyne incognita

## Abstract

**Background:**

Plant-parasitic nematodes developed strategies to invade and colonize their host plants, including expression of immune suppressors to overcome host defenses. *Meloidogyne graminicola* and *M. incognita* are root-knot nematode (RKN) species reported to damage rice (*Oryza sativa* L.) cultivated in upland and irrigated systems. Despite *M. incognita* wide host range, study of the molecular plant - RKN interaction has been so far limited to a few dicotyledonous model plants. The aim of this study was to investigate if the rice cv. Nipponbare widely used in rice genomic studies could be used as a suitable monocotyledon host plant for studying *M. incognita* pathogenicity mechanisms. Here we compared the ability of *M. graminicola* and *M. incognita* to develop and reproduce in Nipponbare roots. Next, we tested if RKNs modulates rice immunity-related genes expression in galls during infection and express the *Mi-crt* gene encoding an immune suppressor.

**Results:**

Root galling, mature females, eggs and newly formed J2s nematodes were obtained for both species in rice cultivated in hydroponic culture system after 4-5 weeks. *Meloidogyne graminicola* reproduced at higher rates than *M. incognita* on Nipponbare and the timing of infection was shorter. In contrast, the infection characteristics compared by histological analysis were similar for both nematode species. Giant cells formed from 2 days after infection (DAI) with *M. graminicola* and from 6 DAI with *M. incognita*. Real-time PCR (qRT-PCR) data indicated that RKNs are able to suppress transcription of immune regulators genes, such as *OsEDS1*, *OsPAD4* and *OsWRKY13* in young galls. Four *M. incognita* reference genes (*Mi-eif-3*, *Mi-GDP-2*, *Mi-Y45F10D.4*, and *Mi-actin*) were selected for normalizing nematode gene expression studies *in planta* and in pre-parasitic J2s. *Meloidogyne incognita* expressed the immune suppressor calreticulin gene (*Mi-crt*) in rice roots all along its infection cycle.

**Conclusion:**

RKNs repress the transcription of key immune regulators in rice, likely in order to lower basal defence in newly-formed galls. The calreticulin Mi-CRT can be one of the immune-modulator effectors secreted by *M. incognita* in rice root tissues. Together, these data show that rice is a well suited model system to study host- *M. incognita* molecular interactions in monocotyledons.

**Electronic supplementary material:**

The online version of this article (doi:10.1186/s12284-014-0023-4) contains supplementary material, which is available to authorized users.

## Background

Root-knot nematodes (RKNs, *Meloidogyne* spp*.*) are one of the most economically damaging genera of plant-parasitic nematodes on horticultural and field crops in all temperate and tropical areas (Trudgill and Blok [2001]). In particular, crops important for tropical countries as coffee, cotton, cowpea, peanut, soybean and rice are highly susceptible to RKNs, including *M. incognita* (Kofoid and White 1919; Chitwood 1949), *M. arenaria* (Neal 1889; Chitwood 1949), *M. javanica* (Treub 1885; Chitwood 1949), and *M. graminicola* (Golden and Birchfield [1968]). *Meloidogyne* spp. are obligate plant parasites that settle in roots and complete their life cycle by feeding from host cells (Williamson and Gleason [2003]). Like other plant and animal parasites, plant-parasitic nematodes developed strategies to invade and colonize their host plants, subvert the host machinery to their own benefit and overcome host defenses (Haegeman et al. 2012; Rosso et al. [2012]; Mitchum et al. [2013]). *Meloidogyne* spp. (juveniles stage J2) usually enter the plant through the apex and the root elongation zone, and then migrate between plant cells to reach the young central cylinder. Recent genomic data showed that *M. incognita* and *M. hapla* (Chitwood, 1949) genomes contain a high number of cell wall degrading enzymes, indicating that the nematode may use a combination of mechanical piercing and cell wall softening to enter and migrate into roots (Abad et al. [2008]; Opperman et al. [2008]; Danchin et al. [2010]). Once going into the differentiating vascular tissues, juveniles become sedentary and initiate nourishing feeding site originated from few parenchyma cells. Concomitantly, neighbouring cells divide causing roots to form knots or swellings. It has been shown that secretions from the nematode are crucial in establishment of the nourishing feeding site within the host root (Bellafiore and Briggs [2010]; Rosso et al. [2012]; Mitchum et al. [2013]). By secreting a number of compounds (including effectors) into root cells, RKNs induce their differentiation into hypertrophied, multinucleate and metabolically active feeding cells, named giant cells (GCs) (Kyndt et al. [2013]). Feeding-site formation enables the parasites to pump large amounts of nutrient solutions from the plant's vascular system. The nematode then goes through two developmental stages (J3, J4) to finally differentiate into an adult female which will lay eggsand new juveniles arising from these eggs will, in turn, start a new reproduction. Depending on the host plant and environmental conditions, the cycle lasts 15-45 days (Triantaphyllou and Hirschmann [1960]; Perry and Moens [2011]). It is critical for the nematode to cope with the host immune responses all along the infection process. One strategy is most likely the release of immune-modulatory effectors that block or interact with the plant basal defense network (Bellafiore and Briggs, [2010]). A series of transcriptome analyses in *Arabidopsis* and tomato have shown that, when a clear induction of the cell primary metabolism is evident, the expression of genes related to the plant immune responses are down-regulated in galls during plant-nematode interactions (Barcala et al. [2010]; Caillaud et al. [2008]b).

Until now, functional analysis of *Meloidogyne* spp*.* effectors has been essentially limited to *M. incognita* and to a lower extent to *M. javanica*, having *A. thaliana* or tomato as host plants. *Meloidogyne incognita* has a wide host range encompassing several hundreds of wild and cultivated plants. It is thus hypothesized that *M. incognita* pathogenicity mechanisms are conserved across plant genera, and even between dicotyledons and monocotyledons (Bellafiore and Briggs [2010]; Rosso et al. [2012]). However, the functional characterization of *M. incognita* effectors in other plant hosts, including monocot species, has been poorly investigated. Among plant species amenable to high-throughput genetic transformation and analysis, rice (*Oryza sativa*) is well-suited as a model monocot and is a crop species of high agronomic value and a RKN host.

*Meloidogyne* species damage upland rice in Asia, West Africa and Latin America with a prevalence of up to 50% (Bridge et al. [2005]). Up to now, nearly all grown *O. sativa* varieties tested are susceptible to *Meloidogyne* spp. infection, even when differences in host response to *M. graminicola* or *M. incognita* infection can be observed (Diomandé [1984]; Bridge et al. [2005]; Prasad et al. [2006]; de Araújo-Filho et al., 2010). Specific resistances to *Meloidogyne* spp. were identified in the African relative species like *O. glaberrima* (Diomandé [1984]; Soriano et al. [1999]) and progenies derived from inter-specific crosses are currently being tested for nematode resistance.

The *M. graminicola* life cycle in rice roots was recently investigated by histopathological analysis in several *O. sativa* and *O. glaberrima* rice varieties (Cabasan et al. [2012]). Analysis of the molecular rice responses to *M. graminicola* infection showed that the hormone-mediated resistance signaling pathways controlled by salicylic acid (SA), jasmonic acid (JA) and ethylene (ET) are repressed soon after infection by *M. graminicola* (Nahar et al. [2011]).

In contrast, the rice - *M. incognita* interaction has been poorly investigated until now and only little histological data was published (Ibrahim et al. [1973]). How the rice host plant copes with various RKN species has been poorly investigated. Therefore, the aim of this study was to investigate if rice could be used as a suitable host plant for studying *M. incognita* pathogenicity mechanisms and plant defense responses. Here we tested the *O. sativa* cv. Nipponbare for *M. incognita* susceptibility, and compared the ability of *M. graminicola* and *M. incognita* to develop and reproduce in roots of this rice cultivar. Next, we tested the hypothesis that the nematode modulates host defense responses in rice plants. We show that *M. incognita* expressed the calreticulin gene (*Mi-crt*) in infected rice roots and that several rice defense genes expression are down-regulated at an early stage of infection when the nematode starts feeding from root cells. Together, these data show that rice can provide an excellent model system to study host-*M. incognita* molecular interactions in monocotyledons.

## Results

### Root galling and reproduction of *M. incognita* in *O. sativa* cv. Nipponbare

#### Reproduction and life cycle duration

**Figure 1:**
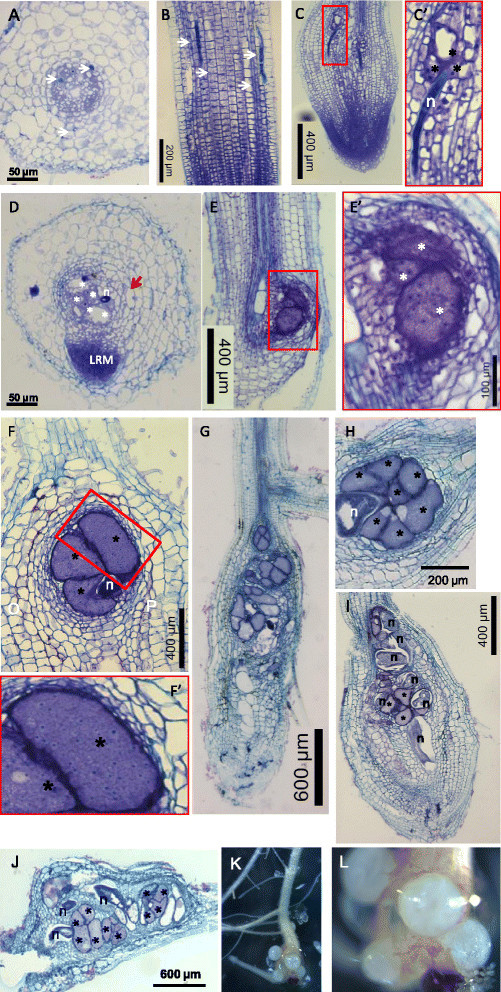
**Cross- and longitudinal- sections (10 μm) of Nipponbare rice root infected by** ***Meloidogyne incognita*** **.** **A-B**: 2 DAI; **C-C'**: 4 DAI; **D**: 6 DAI; **E-E'**: 7 DAI. (LRM: lateral root meristem; n: nematode; asterisk: giant cell). **F-F'**: 15 DAI; **G-H**: 22 DAI; **I**: 35 DAI; **J**: 42 DAI; **K-L**: photographs of a gall with females protruding from the root and egg masses induced by *M. incognita*. (n: nematode; asterisk: giant cell).

Root swellings resulting from one or multiple galls formed within the rice root system were visible around 4 days after inoculation (DAI), and egg-laying females were observed from approximately 22 DAI, with a majority observed after 28 DAI (Figure 1K-L). Freshly hatched juveniles appeared at 35 DAI and continued until 56 DAI. *M. incognita* cycle (J2 to J2) duration on rice cv. Nipponbare was thus estimated to 35 days. Galls were initially formed at the root tips of young plantlets and some infected roots stopped their growth. After 35 DAI, galls were found either at root tips in roots which stopped expanding or were distributed in roots which took over expansion.

#### Histological analysis of rice roots nematode infection

Nematodes entered the roots, preferentially via the root elongation zone migrating via the cortex to the root tip and then migrating up into the vascular cylinder where it induces feeding cells (Figure 1). At 2 DAI, the observation of transverse sections of roots stained with toluidine blue showed that the nematodes were protruded out or were inside the stele (Figure 1A). Nematodes migrated inter-cellularly and no sign of broken or necrotic root cells was observed (Figure 1B). Along the path of migrating nematodes cortical cells presented hypertrophy and asymmetrical shapes most likely induced by the presence of the infecting nematode. No clear sign of binucleate initiating giant cells were observed although signs of feeding cell induction like condensed cytoplasm could be infrequently seen.

At 4 DAI, a number of nematodes were still migrating and caused root swelling. Within the stele a number of nematodes became sedentary and selected vascular parenchyma tissue containing xylem and phloem to induce their feeding site. Acytokinetic nuclear division was then observed in young feeding cells which presented a dense cytoplasm, identified as newly induced GCs close to the nematode head (Figure 1C,C'). Parenchymatic cells of the vascular cylinder, neighboring giant cells (NCs) divided, lost their typical rectangular shape and presented irregular shapes encircling the giant-feeding cells. New xylem and phloem elements also proliferated in the proximity of giant cells. These NCs showed a denser cytoplasm and nuclei became more prominent when closer to GCs (Figure 1C, and for 6 DAI, Figure 1D).

At 6 to 7 DAI, the formation of several feeding sites within the vascular system was observed and some J2 were still found migrating within the stele. Cross sections of galls showed GCs surrounded by dividing cells apparently originated from the vascular cylinder as well as from the root cortex. A number of cortical cells seemed to divide asymmetrically (red arrow in Figure 1D). The induction of lateral roots originating from pericycle cells was also visible situated at the feeding site (Figure 1D). Multinucleation as well as enlargement of GCs was observed at this stage indicating that intense DNA replication was taking place (Figure 1E, E').

At 15 to 21 DAI (Figure 1F-G), it could be evidenced that most root swellings contained multiple nematode feeding sites (NFS) (Figure 1G). Each NFS contained in average 5 to 7 GCs. GCs appeared more frequently embedded in the vascular cylinder, presented thickened cell walls and contained a dense cytoplasm with a large number of nuclei (per 10 μM section; Figure 1F,F'). Nematodes at this stage (15 to 21 DAI) varied between parasitic J2 and maturing females. Therefore, histological observations at these time points suggest that nematodes underwent molts (J2-J3-J4-young females). GCs at this stage are mature, implying that nuclei division and enlargement ended.

At 32 to 42 DAI, a number of females started egg-laying and due to their large size they were lost during sectioning (Figure 1G-J). GCs devoid of cytoplasm might be due to nematodes which stopped feeding after reproduction or that died.

### Root galling and reproduction of *M. graminicola* in *Oryza sativa* cv. Nipponbare

#### Histological analysis of rice roots nematode infection

**Figure 2:**
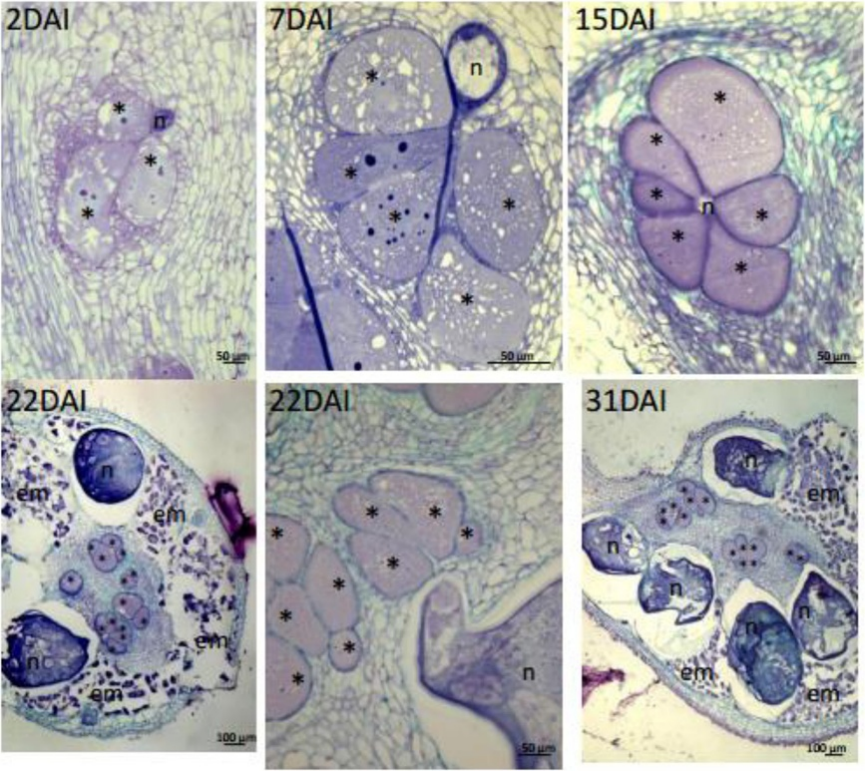
**Cross-sections (10 μm) of Nipponbare rice roots infected by** ***Meloidogyne graminicola*** **obtained at 2, 7, 15, 22 and 31 days after nematode infection (DAI).** (n: nematode; asterisk: giant cell; em: egg masses).

*Meloidogyne graminicola* penetrated rice roots via the root elongation zone, downwards to the root meristem and upwards to the vascular cylinder as observed for *M. incognita*. Examination of roots at 1 DAI confirmed the presence of nematodes in the cortex. Roots showed swellings very early at 2 DAI and galls were obvious at 4 DAI. Histological analysis showed that NFS were visible in swollen root tips from 2 DAI (Figure 2). They were formed of 3-4 young giant cells and located within the vascular cylinder where phloem, xylem and neighboring parenchymatic cells proliferated at the surroundings. At 4 to 7 DAI young GCs contained a dense cytoplasm, presented several nuclei and thicked cell walls compared to walls of vascular parenchyma cells (Figure 2). At 15 DAI giant cells presented multiple enlarged nuclei (Figure 2). Cells in direct contact or very close by GCs contained a dense cytoplasm. Often more than one NFS was observed per root swelling. At 22 DAI mature galls contained egg-laying females which remained embedded in the root tissues exterior to the gall proper (Figure 2). Around 31 DAI GCs were filled with cytoplasm and nuclei of various shapes often clustered. In some galls, some GCs appeared degraded.

#### Reproduction and cycle duration

Stage 2 juveniles became parasitic around 4 to 7 DAI and appeared swollen at this stage. At 15 DAI nematode had undergone molts of J2 to J3 and J4 non-feeding finally reaching the female stage (7 to 15 DAI) and egg laying adult females were observed from 18 to 22 DAI. Female bodies and egg masses were embedded within the gall tissues (cortex). At 31 DAI, eggs laid by mature females hatched and most likely infected roots within the same plant.

### Nematode genes expression in rice roots

A time course experiment with *O. sativa* cv Nipponbare infected with *M. incognita* was applied to study the *in planta* nematode gene expression. Swollen root tips and galls located outlying from the root apex, were collected at 6, 10 and 20 DAI and RNA was extracted. For each time points, at least 35 plants were inoculated. The experiment was repeated in triplicate.

Transcript accumulation of 5 genes (*Mi-csq-1*, *Mi-eif-3*, *Mi-GDP-2*, *Mi-Y45F10D.4*, and *Mi-actin*) putatively constitutively expressed in *M. incognita* was measured in root samples collected at different times after infection. In all three time-course experiments tested, the nematode genes transcript levels increased in root samples all along infection (Additional file 1: Table S1).

**Figure 3:**
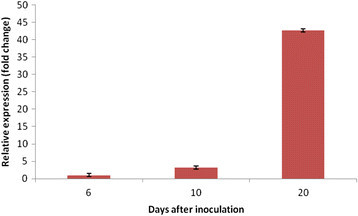
**Relative expression average of** ***M. incognita*** **constitutive genes in infested rice** ***O. sativa*** **cv. Nipponbare roots.** Gene expression was measured by reverse transcription-quantitative polymerase chain reaction in plants infested with *M. incognita* at different time points after treatment. Bars are the mean values (± standard error of the mean [SEM]) of *Mi-csq-1*, *Mi-eif-3*, *Mi-GDP-2*, *Mi-Y45F10D.4*, and *Mi-actin* genes expression data (Cq) in three independent biological replicates, with 35 plants per condition. (*n=5; 3, each contained 35 plants pooled*).

Figure 3 shows the averaged expression levels (as defined by Cq values) of the 5 *M. incognita* genes here analyzed in infected root tissues. On average, a 42-fold (deltaCq = 5.4) difference was found in the transcript accumulation between samples collected at the earliest (6 DAI) and latest (20 DAI) time tested after inoculation with *M. incognita* (Figure 3). *Mi-actin* transcript levels were the most elevated in root samples as compared to other genes tested (Additional file 1: Table S1) and exhibited a 128-fold change accumulation during the time course after infection. The stability of each *M. incognita* gene expression across samples was analyzed using RefFinder (Xie et al. [2012]) in order to select the most reliable genes for using them as *M. incognita* reference genes for qPCR gene expression analysis in rice tissues. Except *Mi-csq-1*, all genes showed good stability across samples (Additional file 2: Figure S1). The *Mi-actin* gene showed the most stable expression pattern (lowest geometric mean) and was used to further normalize *Mi-crt* expression levels.

In parallel, the *Os-actin* transcripts were quantified to verify the amount of rice transcripts in galls. When compared to other rice reference genes, *Os-actin* showed stable expression patterns in galls and roots of *O. sativa* cv. Nipponbare plants (data not shown). Contrary to the enhanced *Mi-actin* transcript accumulation, *Os-actin* transcripts decreased during the infection course, mainly after 10 DAI (an average difference of 2 cycles between the 6- and 20-DAI samples) (Additional file 2: Figure S2). These data suggest that nematode representation is increasing in the biological material collected from infected roots along the time-course tested. This is in accordance with the histological data showing that the nematode body size dramatically increased inside galls. These data then indicate that the RNA extraction procedure used for the mixed plant-nematode samples was efficient for the purification of the total RNAs from both organisms.

**Figure 4:**
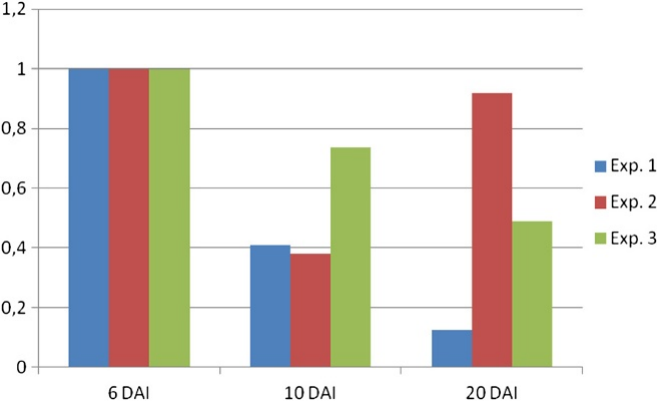
**Relative expression of the** ***Mi-crt*** **gene in** ***M. incognita*** **-infested rice** ***O. sativa*** **cv. Nipponbare roots after 6, 10, and 20 dai.** Gene expression was measured by reverse transcription-quantitative polymerase chain reaction in plants infested with *M. incognita* at different time points after treatment. The *Mi-crt* data were normalized to the *Mi-actin* data and *Mi-crt* gene expression in infected-rice samples was further calculated relative to the 6 DAI sample. Data presented are means (± standard error of the mean [SEM]) expression in three independent biological replicates, with 35 plants per condition (*n=3, each contained 35 plants pooled*).

Expression of the *M. incognita* effector gene *Mi-crt* was followed in root samples at 6, 10 and 20 DAI. The *Mi-crt* data were normalized to the *Mi-actin* data and *Mi-crt* gene expression in infected-rice samples was further calculated relative to the 6 DAI sample (Figure 4). *Mi-crt* transcripts were as abundant as *Mi-actin* transcripts, and accumulated at the three infection stages tested. In addition, the *Mi-crt* expression data were not found to significantly differ between 6 DAI, compared to 10 and 20 DAI, as expected from Jaubert et al. ([2005]) in *M. incognita* - *A. thaliana* interaction.

### Rice defense genes expression

To study the rice defense responses against *Meloidogyne* spp. root samples were collected from Nipponbare plants inoculated with *M. graminicola* or *M. incognita* at 2 and 6 DAI, respectively. These time points correspond to the formation of nutrient feeding sites observed in rice roots for each nematode species, respectively. For each time point chosen, a series of 90 plants were inoculated, and root samples were collected and pooled together. The experiment was repeated in duplicate. Non-inoculated plants served as control at the time of inoculation.

A series of genes involved in the rice immune responses were selected, including those from signaling, salicylic acid (SA)- and jasmonic acid (JA)-dependent resistance signaling pathways (Delteil et al. [2010]). We chose *OsMAPK6*, *OsMAPK5a* and *OsMAPK20* for early signaling (phosphorylation cascades in PTI and ETI), *OsAOS2* (JA pathway), *OsEDS1* and *OsPAD4* (SA-dependent resistance), *OsRAC1* (oxidative burst), *OsNIH1*, and *OsWRKY13* (positive transcriptional regulators of defense genes). Gene expression was normalized to the rice *Os-actin* used in Tao et al. ([2009]) and Delteil et al. ([2011]).

**Figure 5:**
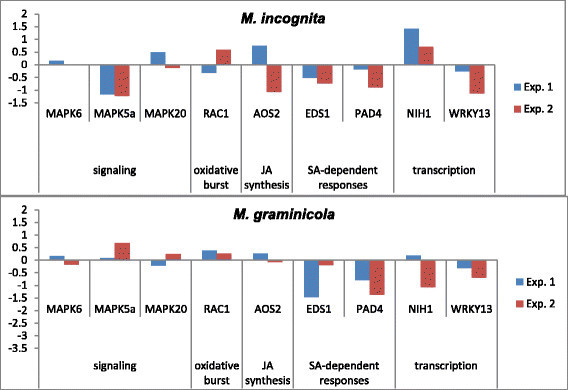
**Early regulation of defense-related genes in** ***O. sativa*** **cv. Nipponbare roots during** ***Meloidogyne*** **infection.** Gene expression was measured by reverse transcription-quantitative polymerase chain reaction in plants infested with *M. graminicola* at 2 DAI or with *M. incognita* at 6 DAI. Gene expression was normalized to *Os-actin*. Bars represent the log2 values of the ratio of the mean transcript levels for inoculated vs. non-inoculated plants from two technical replicates. Two independent biological replicates were carried out, with 90 plants per condition.

Analysis of gene expression in rice roots challenged with *Meloidogyne* spp. showed that the majority of these genes, except for *NIH1* that was induced two-fold at this infection stage for *M. incognita*, were not induced or down-regulated in the early time points after inoculation (Figure 5) as compared with non-inoculated control plants. This was particularly evident for *OsEDS1*, *OsPAD4* and *OsWRKY13* genes tested that were down-regulated from two- to three-fold at 2 DAI in *M. graminicola* and 6 DAI in *M. incognita*.

## Discussion

In this study, we showed that *M. incognita* is able to develop and reproduce in *O. sativa* cv. Nipponbare. Root galling, mature females, eggs and freshly hatched J2s were obtained in hydroponic culture system after 4-5 weeks. *Meloidogyne incognita* is naturally found associated to rice in rainfed growing systems but the impact of this RKN species on rice cultivation is lower than *M. graminicola* that is remarkably well adapted to flooded conditions (Fortuner and Merny, [1979]; De Waele and Elsen, [2007]). In this study, we observed that *M. graminicola* reproduced at higher rates than *M. incognita* on Nipponbare and the timing of infection was shorter although the infective behaviour and feeding site morphologies were similar for both nematode species. Juveniles penetrated the root elongation zone and tips, and established in the stele where they induced the formation of nutrient providing feeding sites. Giant cells were already visible at 2 DAI with *M. graminicola* and only at 6 DAI with *M. incognita*. These giant-feeding cells presented walls that thickened during their development exhibiting apparent cell wall ingrowths and interruptions indicative to be plasmodesmata. Development of galls induced by *M. incognita* as well as by *M. graminicola* in *O. sativa* cv. Nipponbare involves hypertrophy of vascular parenchyma cells ending up in a small number of giant cells per gall (up to 7). Concomitantly, hyperplasy is observed in cells neighboring the giant-feeding cells and xylem and phloem cells. A small number of cells around the gall proper also undergo to some extent hypertrophy. In addition, other feeding sites form in the root vicinity meaning that each root swelling may contain multiple galls. Highnumber of feeding sites (or galls) is noteworthy in each root swelling caused by *M. graminicola* as recently reported by Cabasan et al. ([2013]). The presence of multiple galls suggests that during this susceptible interaction hyperplasy might be further induced in the root surrounding area. Therefore, it is tempting to say that multiple gall formation might be facilitated by the presence of neighboring galls. Otherwise, simply multiple galls form close to each other because several juvenile nematodes penetrate concurrently. A gall induced by *M. incognita* in rice roots stays mostly confined to the vascular cylinder whereas apparently galls induced by *M. graminicola* involve a small number of cells outside of the vascular tissue. This is in agreement with galls induced in the dicotyledon model host, *Arabidopsis thaliana* where galls are strictly confined to the vascular tissue (de Almeida Engler et al., [1999]). Galls in *Arabidopsis* do not induce proliferation of endodermal tissue layer, as revealed by lignin stain of galls delimited the feeding site (de Almeida Engler, unpublished data). Number of outer tissue layers of rice galls excluded from the vascular cylinder at different root regions varies from 5 to around 10 cell layers, but this could simply reflect the root region that the gall develops. Therefore, there it is not yet clear that cells outside the vascular cylinder are part of the gall induced by *M. graminicola*. Initial phase of gall development shows giant cells which undergo the first nuclear division accompanied by increase in cytoplasm density. Concomitantly, parenchymatic cells neighboring giant cells (NCs) divide and lose their typical rectangular shape and become more rounded with various shapes encircling the giant-feeding cells. These NCs show a denser cytoplasm and nuclei become more prominent when encircling giant cells, suggesting some kind of cellular communication between giant cells and NCs. The presence of plasmodesmata devoid of callose deposition has been demonstrated for galls induced by root-knot nematodes in *Arabidopsis* roots by Hofmann et al. ([2010]). The presence of lateral root meristems in the gall region was also observed during gall development suggesting local auxin accumulation.

Once galls mature nematode developed into the classical J2-J3-J4-female phases and egg-laying females were observed at 22 DAI with *M. graminicola* and at 32 DAI with *M. incognita*. A conspicuous feature between both *Meloidogyne* species was that *M. incognita* lays eggs outside of the roots whereas *M. graminicola* eggs remain embedded within the root tissues, this latest explaining why *M. graminicola* is a RKN species successful in infecting rice grown in irrigated or flooded systems (Fortuner and Merny, [1979]; Prot and Matias, [1995]; De Waele and Elsen, [2007]). *Meloidogyne graminicola* female body size at 31 DAI was similar to the size of *M. incognita* females. Evidently, egg laying within the root cortex by *M. graminicola* is thus a specific adaptation for the aquatic survival and infection ability. In addition, the fact that *M. graminicola* eggs are kept within the roots while *M. incognita* eggs are extruded out of the root tissue might represent an adaptation to their hosts: to water submersed root systems, or to the soil environment, respectively. Understanding the genetic basis of this adaptation might therefore be a novel clue in nematode parasitism knowledge in plants with submersed roots versus soil grown root systems.

Importantly, data presented here indicate that both RKN species are able to suppress rice basal defence genes expression at early stages after infection. We hypothesize that RKN repress the transcription of key immune regulators in order to lower the basal defence. This is in accordance with Nahar et al. ([2011]) and Ji et al. ([2013]) who reported that rice cv Nipponbare defense genes expression was repressed in roots and giant cells early upon infection with *M. graminicola*. Already at 1 DAI, the mRNA levels of four immune-related genes tested (*OsWRKY45*, *OsPR1b*, *OsEin2b*, and *JiOsPR10*) were significantly attenuated in gall tissues (Nahar et al., [2011]). Here we show that the same occurred in rice galls infected by *M. graminicola* at 2 DAI, or by *M. incognita* at 6 DAI, when the nematode had started feeding and that first nutrient feeding sites have been established (Figures 1 and 2). Eight genes involved in the rice immune responses were tested, including those from cell signaling, SA- and JA-dependent resistance signaling pathways. When overexpressed in rice plants, *OsAOS2*, *OsRAC1*, *OsNIH1*, *OsWRKY13* genes presented enhanced resistance levels to the fungus *Magnaporthe oryzae*, causing rice blast disease and the bacteria *Xanthomonas oryzae* pv. *oryzae* causing bacterial blight (Delteil et al. [2010]). *EDS1* (Enhanced Disease Susceptibility 1) and *PAD4* (Phytoalexin Deficient 4) play a key role in *A. thaliana* R-protein-triggered and basal resistance to invasive biotrophic and hemi-biotrophic pathogens (Falk et al. [1999]; Jirage et al. [1999]). EDS1 and PAD4 are intracellular proteins homolog to lipases that can interact and form different protein complexes. In response to infection, EDS1 and PAD4 activate SA production and signaling, and also mediate antagonism between the JA and ET defense response pathways (Rietz et al. [2011]; Wiermer et al. [2005]). In the same way, *OsWRKY13* serves as a node of the antagonistic crosstalk between SA- and JA-dependent pathways in pathogen-induced defense responses. There are more than 100 *WRKY* transcription factors in the rice genome (Wu et al. [2005]), and many are involved in rice innate immune responses, including *OsWRKY03*, *OsWRKY71*, *OsWRKY13*, *OsWRKY45* (Qiu et al. [2009]; Liu et al. [2013]). *OsWRKY13* mediates disease resistance to bacterial blight and fungal blast through activation of SA-dependent pathways and suppression of JA -dependent pathways (Qiu et al. [2009]). The present study strongly suggests that successful *M. incognita* or *M. graminicola* attack suppresses SA signaling in rice, as revealed by the down-regulation of *OsEDS1*, *OsPAD4* and *OsWRKY13* genes in newly-formed galls. This result is in accordance with Nahar et al. ([2011]) who observed down-regulation of genes involved in SA/JA/ET signaling in rice cv. Nipponbare challenged with *M. graminicola*. However, rice plants impaired in SA biosynthesis (expressing the *Pseudomonas putida* salicylate hydroxylase *NahG* gene) were only slightly more susceptible to nematode infection, pointing to a positive but minor role for SA in rice defense against *Meloidogyne*. In tomato, *NahG* plants that still produce minimal amounts of SA did not show increased susceptibility to *Meloidogyne* indicating that low levels of SA might be sufficient for basal resistance to root-knot nematodes (Bhattarai et al. [2008]). In addition, successful development of *Meloidogyne* in tomato roots involves the repression of pathogenesis-related (PR) genes associated to SA-dependent systemic acquired resistance (SAR) (Molinari et al. [2013]). Conversely, the JA/ET signalling pathway seems to play a major role in basal defense to nematodes (Bhattarai et al. [2008]; Nahar et al. [2011]). When exogenous ethephon and methyl jasmonate were supplied to rice plants, *M. graminicola* was less effective in counteracting root defense pathways. Here, the *OsAOS2* gene, which codes the first enzyme for JA biosynthesis pathway, was not induced in response to nematode infection at the time studied. This suggests that the plant did not perceive nematode attack or that proper signalisation was impaired.

Mitogen-activated protein kinase (MAPK) cascades are activated in plants during responses to pathogens and mediate intracellular stress responses, including reactive oxygen species (ROS) production, cell death, and activation of PR gene expression. The *OsMAPK6* cascade plays an important role in both pathogen-associated molecular pattern (PAMP)-triggered immunity (PTI) and effector-triggered immunity (ETI) in rice (Liu et al. [2013]). *OsMAPK6*, *OsMAPK5a* and the rice small GTPase *OsRac1* act together to regulate plant defense responses to pathogens or to pathogen-derived elicitor signaling in rice (Lieberherr et al. [2005]; Liu et al. [2013]). In this study, *OsMAPK5a* was down-regulated in rice galls, and more specifically in response to *M. incognita* infection. Recently, *OsMAPK5* was shown to negatively modulate PR genes expression in rice, such as *PR1* and *PR10* (Xiong and Yang [2003]; Seo et al. [2011]). When *OsMAPK5a* is knocked-down in rice, plants appear more resistant to fungal *(M. oryzea)* and bacterial *(Burkholderia glumae)* pathogens (Xiong and Yang, [2003]). However, *OsMAPK5a* transcription has been reported to be activated in response to *M. oryzae* (for both virulent and avirulent isolates) few hours after inoculation, but it is further down-regulated until 3 DAI (Delteil et al. [2012]). Here it is thus not clear whether *OsMAPK5a* would also act as a negative regulator of rice defense responses to nematodes.

It is now known that the local suppression of host defense signaling observed after nematode infection is the result of virulence effectors secreted into the host plant to facilitate infection (Haegeman et al. [2012]; Mitchum et al. [2013]). *Meloidogyne* spp. potentially secrete hundreds of proteins in its host (Bellafiore et al. [2008]). In this study, we showed that *M. incognita* expressed the calreticulin *Mi-CRT* gene all along its infection cycle in Nipponbare roots. In *A. thaliana*, Mi-CRT is secreted *in planta* throughout parasitism (Jaubert et al. [2005]) and plays a role as immune-modulator in the suppression of plant basal defenses (Jaouannet et al. [2013]). It is thus expected that Mi-CRT play a similar role in interfering with the plant immune system PTI in distantly related hosts. Calreticulins are highly conserved calcium-binding proteins in plants and animals that act as Ca^2+^ - binding chaperones, regulating Ca^2+^ storage and signalling in the cell. But, how Mi-CRT contributes to the infection process of the nematode remains unknown and should be further investigated in particular during rice infection.

## Conclusions

Our data demonstrate that the *M. incognita*-rice pathosystem may be a novel additional model system to dissect the complex cellular and molecular nematode interactions with monocotyledonous host plants. A benefit of the *Meloidogyne*-rice pathosystem over other plant-nematode models is the specific resistance identified in the African relative species *O. glaberrima* (Soriano et al. [1999]). The rice, *O. sativa* and *O. glaberrima* - *Meloidogyne* spp*.* interactions thus may serve as a model to understand compatible and incompatible plant- nematode interactions respectively and to elucidate the molecular mechanisms developed by these parasites to infect their monocotyledonous host plants.

## Methods

### Biological material

*Meloidogyne incognita* isolate 1 (race 1, USA; Li et al. [2007]) was propagated from greenhouse-grown tomato plants (*Solanum lycopersicum* L*.* cv. Naine). Second-stage juvenile (J2) nematodes were hatched from sterilized eggs as described (Bellafiore et al. [2008]). Eggs were hatched at room temperature for 5 days, and J2 worms were allowed to crawl through six Kimwipe tissue layers in water with 100 mg/l streptomycin. The population of *M. graminicola* used in all experiments was originally collected from Laurel (Batangas, Philippines) and cultured on *Oryza sativa* cv. IR64. Eggs were extracted from infected roots by shaking *M. graminicola*-infected roots in bleach 0.7% for 5 min and mixing them in a "blender" for 5 times during 1s. Eggs were collected onto a 25 μm mesh and were hatched at room temperature. Only J2 nematode populations collected after a maximum of 96 h were used as inoculums.

### Nematode inoculation assays on rice plants

*Oryza sativa* subsp*. japonica* cv. Nipponbare seeds were germinated on sand wetted with Hoagland ¼ solution for 7 days and then transferred to tubes containing 10 g SAP substrate (Reversat et al. [1999]) wetted with Hoagland ¼ solution (KNO_3_ 5 mM; KH_2_PO_4_ 1 mM; Ca(NO_3_)_2_ 5 mM; MgSO_4_ 2 mM; 25 mg iron; trace element). Rice plants were maintained in a growth chamber under controlled conditions at 26°C/24°C day/night temperature, under a 14 h day⁄ 10 h night light regime (60 μmol m^-2^ s^-1^ illumination) and 78% relative humidity. Three days after transplanting into SAP (Sand and Absorbent Polymer) substrate (Reversat et al. [1999]), the plants were inoculated with 1 ml water containing 400 freshly hatched stage J2 juveniles of *M. graminicola* or *M. incognita*. One day after inoculation (DAI) rice plants were transferred to a 15 ml hydroponic culture system with Hoagland ¼ solution (Reversat et al. [1999]) to synchronize the infection process.

### Histopathology study

Infected roots were harvested at 1, 2, 4, 7, 15, 22, 31, 35 and 42 DAI, carefully washed and immediately placed in freshly prepared fixative (2% paraformaldehyde - 1% glutaraldehyde - 1% cafein (Sigma-Aldrich) in 0.5 × phosphate buffer (Sigma-Aldrich). Root tips (1 cm segment) or when visible, galls were excised from each plant, fixed for 15 h in PFA, dehydrated for 1 h in each ethanol dilution (once 50% and twice 70% vol/vol) and embedded in the epoxy resin Technovit 7100 (Kulzer Friedrichsdorf, Germany) according to Pegard et al. ([2005]). Blocks containing galls of different time points were sectioned (10 μm), mounted in 90% glycerol and microscopically observed under UV light (UV filter set A2, Zeiss AXIO Imager). The same sections were subsequently stained (3 min at room temperature) with 0.05% toluidine blue in 0.1 M sodium phosphate buffer, pH 5.5. Images were taken with an Axiocam digital camera (Leica microscope) with standard bright-field optics.

### RNA extraction and cDNA synthesis

Root tips were excised from infected and non-infected rice plantlets, immediately frozen in liquid nitrogen and kept at -8°C until use. Total RNA was extracted from rice root samples using the RNeasy Plant kit (Qiagen, France), with addition of an on-column DNase I digestion. For quantification, the absorbance from 1 μL RNA samples were measured using the NanoDrop ND-1000 spectrophotometer (NanoDrop Technologies), and 1% agarose gel was run to visualize the quality of the RNA. First-strand cDNAs were synthesized from 1 μg of total RNA in 20 μl final volume, using Omniscript RT kit (Qiagen) and oligo-dT(18)-MN primer (Eurogentec, Angers, France) following the manufacturer's instructions.

### Reference genes selection in *M. incognita*

Candidate reference genes of *M. incognita* to normalize RT-qPCR studies were selected based on a study on the model nematode *Caenorhabditis elegans* (Hoogewijs et al. [2008]). The *M. incognita* ortholog genes were searched in the genome (http://www6.inra.fr/meloidogyne_incognita), and specific primers were designed for RT-qPCR. Only the primers designed for *Mi-csq-1*, *Mi-eif-3C*, *Mi-gpd-2*, *Mi-Y45F10D.4*, and *Mi-actin* genes displayed correct amplification efficiency and specificity. Transcript accumulation of candidate genes was detected in rice roots after infection with *M. incognita*. The stability of each *M. incognita* gene expression during rice infection was analyzed using RefFinder (Xie et al. [2012]; http://www.leonxie.com/referencegene.php) and the most reliable gene was selected to normalise RT-qPCR data on *M. incognita* gene expression analysis in rice tissues.

### Primer design and selection

Specific primers were designed from the *O. sativa* or *M. incognita* cDNA sequences using the Beacon Designer 5.0 software (Premier Biosoft International, Palo Alto, CA, USA), with melting temperatures (Tm) of 58 ± 5°C, primers lengths of 18 to 25 bp, and amplicon lengths of 75 to 200 bp. Primers were designed from the 3′ region of the gene to ensure gene specificity. For each primer pair, a preliminary real-time assay was performed on *O. sativa* or *M. incognita* pure and mixed cDNAs samples to evaluate the amplification of non-specific products or primer dimmer artefacts. The primer efficiency was experimentally tested with the LinRegPCR programme developed by Ramakers et al. ([2003]) which uses a linear regression analysis of fluorescence data from the exponential phase of PCR amplification to determine amplification efficiency (E). The specificity of PCR products was checked by melting curve analyses and only primers sets producing a single sequence-specific peak in the dissociation curve were used.

### Real-time quantitative PCR assays of gene expression

Primers listed in Table 1 were synthesized by Eurogentec and used at 200 nM final concentration. Quantitative RT-PCR was performed using the Stratagene MX3005P with MxPro v 3.00 software for Comparative Quantitation (Stratagene, La Jolla, CA). Quantitative PCR was carried out on 1.25 ng cDNA in a 15 μL amplification mixture containing MESA BLUE Master Mix Plus for SYBR Assay low ROX (Eurogentec, Belgium). The cycling conditions comprised 10 min polymerase activation at 95°C followed by 40 cycles at 95°C for 15 sec, 55°C for 20 sec and 72°C for 40 sec. Following cycling, the melting curve was determined in the range 55°-°95°C, with a temperature slope of 0.01°C/sec. Each assay was conducted in duplicates and included a no-template control.

**Table 1:** **Primers used for reverse transcription-quantitative polymerase chain reaction gene expression studies**

Name	Gene	Forward	Reverse primer
*Mi-CRT (calreticulin)*	GenBank: AAL40720	GGCTCTGTTGGTATTGACATC	CTTGCGTTCTTCCTCATCTGC
*Mi-actin*	Minc06769*	GCTTTGCTATGTTGCTTTGG	TGTAAGAAGTCTCGTGAATACC
*Mi-csq-1*	Minc11275*	TGATTATTTACAGGAAATGTTTGG	TAGGGTCGTCTAAATTTAATTGG
*Mi-eif-3C*	Minc06181*	AAATTCTTTCGTAGTGCTGTTTC	AAATCTTGTCGTCCTAATGGC
*Mi-gpd-2*	Minc12412a*	AAGCCGTTCTTTCTTTGTATG	AAGCATAACCTTCATAGATTGG
*Mi-Y45F10D.4*	Minc08763*	CAAAGATGATCCCACAATAGG	AAAGTTTTGAATTTGGCATCG
*Os-actin (actin-1)*	RAP-DB: Os03g0718100	CTCTCAGCACATTCCAGCAG	AGGAGGACGGCGATAACAG
*OsAOS2*	RAP-DB: Os03t0225900	GCGAGAGACGGAGAACCC	CGACGAGCAACAGCCTTC
*OsMAPK6*	RAP-DB: Os06g0154500	GATACATTCGCCAACTTCC	CAGTGATGCCAGGTAAGG
*OsMPAK5a*	RAP-DB: Os03g17700	GTCTGCTCCGTGATGAAC	TGATGCCTATGATGTTCTCG
*OsMPAK20*	RAP-DB: Os01t0629900	TCAACTCCAATTCCTGCCAAG	AACAACTCTTCCTGGTCTTGC
*OsNH1 (NPR1)*	RAP-DB: Os01t0194300	AGAAGTCATTGCCTCCAG	ACATCGTCAGAGTCAAGG
*OsRAC1*	RAP-DB: Os01t0229400	GCTTCTTCCATAATAACAACG	AGTTTCTTTCTGGTTACATCC
*OsEDS1*	RAP-DB: Os09t0392100	CAGGAGAGGCAGTGTTAATCAG	GCAAGCGGAGTAAGTGGTATG
*OsPAD4*	RAP-DB: Os11t0195500	TCAGAGGCAAGGCAGTAGTG	ACCGCTCACGCAGGATAG
*OsWRKY13*	RAP-DB: Os01t0750100	GCCAGCGGAGAACGAATC	CTCCTCCTGCTTCACAACC

*http://www6.inra.fr/meloidogyne_incognita.

Baseline and threshold values were automatically determined for all plates and genes using the MxPro software ver. 4.1.0.0 (Stratagene). In order to ensure comparability between data obtained from different genes, in each run all samples were in a same plate.

### Data analysis

Analysis of RT-qPCR fluorescence data with LinRegPCR determined E values for each reaction. Individual Cq values were considered as valid only if the amplification parameters passed all quality checks. For the relative gene expression in rice and nematode samples, data were analyzed using the MxPro software package to obtain the relative expression levels of rice genes. For each sample, the mean Cq value was calculated based on Cq values of both replicates. *Mi-crt* gene expression was normalized to the *Mi-actin* gene (Table 1). *Os-* genes expression was normalized to the *Os-actin* gene (Table 1). Based on the comparative Ct method, data are either expressed as fold changes to calibrator average or as log (base 2) fold changes to calibrator average.

## Authors' contributions

Conceived and designed the experiments: SB, DF. Performed the experiments: NVP, SB, ASP, RH, AB, AA, IM, DF. Analyzed the data: NVP, ASP, RH, JA, IM, DF. Wrote or proofread the paper: DF, JA, PG, ASP, SB, NVP. All authors read and approved the final manuscript.

## Additional files

## Electronic supplementary material

Additional file 1: Table S1.:*Meloidogyne incognita* gene expression in infested rice *Oryza sativa* cv. Nipponbare roots and juvenile stage 2 (J2) sample. (DOC 35 KB)

Additional file 2: Figure S1.: Geomean of ranking values of genes reference used. Transcript accumulation of candidate genes was detected in rice roots after infection with *M. incognita*. The stability of each *M. incognita* gene expression during rice infection was analyzed using RefFinder (Xie et al. [2012]; http://www.leonxie.com/referencegene.php). **Figure S2.** Relative expression of the *Os-actin* gene in *M. incognita*-infested rice *O. sativa* cv. Nipponbare roots. Gene expression was measured by reverse transcription-quantitative polymerase chain reaction in plants infested with *M. incognita* at different time points after treatment. Data presented are mean values of two technical replicates. Three independent biological replicates were carried out, with 35 plants per condition (*n = 3, each contained 35 plants pooled*). (DOCX 55 KB)

Below are the links to the authors’ original submitted files for images.

Authors’ original file for figure 1

Authors’ original file for figure 2

Authors’ original file for figure 3

Authors’ original file for figure 4

Authors’ original file for figure 5

## Abbreviations

RKN: Root-knot nematode
DAI: Days after inoculation
GCs: Giant cells
NCs: Neighboring giant cells
NFS: Nematode feeding site
J2: Juvenile stage 2
Cq: Cycle quantification
SA: Salicylic acid
JA: Jasmonic acid
ET: Ethylen
PR: Pathogenesis-related
SAR: (SA)-dependent systemic acquired resistance
MAPK: Mitogen-activated protein kinase
PTI: PAMP-triggered immunity
PAMPs: Pathogen-associated molecular patterns
ETI: Effector-triggered immunity
