# Supplementary material for: Meloidogyne incognita - rice (Oryza sativa) interaction: a new model system to study plant-root-knot nematode interactions in monocotyledons
Source: Rice (N Y). 2014 Sep 22;7:23. doi: 10.1186/s12284-014-0023-4 (PMC4884005; doi:10.1186/s12284-014-0023-4)
Supplement: Supplementary file 1 — Additional file 1: Table S1.:Meloidogyne incognita gene expression in infested rice Oryza sativa cv. Nipponbare roots and juvenile stage 2 (J2) sample. (DOC 35 KB) [file 12284_2014_23_MOESM1_ESM.doc]

**Additional file 1: Table S1.** ***Meloidogyne incognita* gene expression in infested rice *Oryza sativa* cv. Nipponbare roots and juvenile stage 2 (J2) sample.**

Gene expression was measured by reverse transcription-quantitative polymerase chain reaction in plants infested with *Meloidogyne incognita* at 6, 10, and 20 days after inoculation (DAI). Data resented are mean Cq values of two technical replicates. Three independent biological replicates were carried out, with 35 plants per condition (*n=3, each contained 35 plants pooled*).

| Samples | csq1 | eif3 | GDP2 | y45f1 | actin |
| --- | --- | --- | --- | --- | --- |
| 6 DAI1 | 36,4 | 33,42 | 32,78 | 36,6 | 24,56 |
| 6 DAI2 | 35,87 | 34,59 | 32,66 | 36,56 | 24,73 |
| 6 DAI3 | 35,61 | 33,68 | 31,95 | 36,51 | 24,15 |
| 10 DAI1 | 35,84 | 32,79 | 29,81 | 34,44 | 21,86 |
| 10 DAI2 | 37,16 | 32,92 | 29,52 | 34,69 | 21,75 |
| 10 DAI3 | 37,30 | 32,54 | 29,29 | 34,23 | 20,94 |
| 20 DAI1 | 34,24 | 29,67 | 24,99 | 30,65 | 17,64 |
| 20 DAI2 | 32,71 | 29,00 | 25,24 | 31,25 | 17,35 |
| 20 DAI3 | 33,23 | 29,55 | 25,22 | 30,38 | 17,73 |
| J2 | 26,35 | 28,48 | 20,76 | 29,31 | 14,82 |
