# Supplementary material for: Meloidogyne incognita - rice (Oryza sativa) interaction: a new model system to study plant-root-knot nematode interactions in monocotyledons
Source: Rice (N Y). 2014 Sep 22;7:23. doi: 10.1186/s12284-014-0023-4 (PMC4884005; doi:10.1186/s12284-014-0023-4)
Supplement: Supplementary file 2 — Additional file 2: Figure S1.: Geomean of ranking values of genes reference used. Transcript accumulation of candidate genes was detected in rice roots after infection with M. incognita. The stability of each M. incognita gene expression during rice infection was analyzed using RefFinder (Xie et al. [2012]; http://www.leonxie.com/referencegene.php). Figure S2. Relative expression of the Os-actin gene in M. incognita-infested rice O. sativa cv. Nipponbare roots. Gene expression was measured by reverse transcription-quantitative polymerase chain reaction in plants infested with M. incognita at different time points after treatment. Data presented are mean values of two technical replicates. Three independent biological replicates were carried out, with 35 plants per condition (n = 3, each contained 35 plants pooled). (DOCX 55 KB) [file 12284_2014_23_MOESM2_ESM.docx]

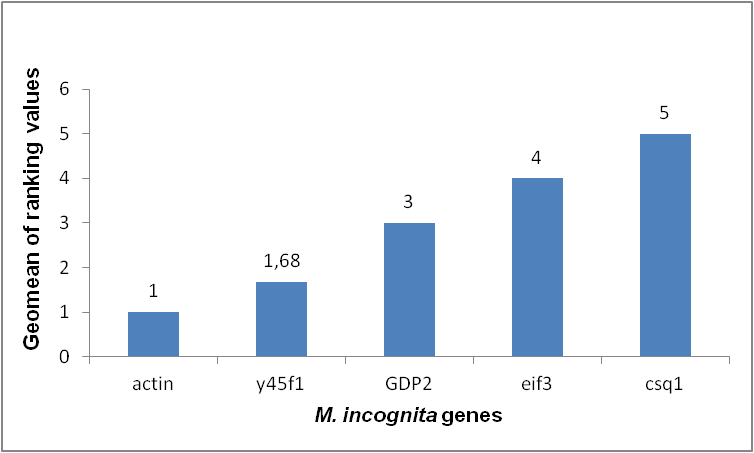


**Additional file 2: Figure S1.** **Geomean of ranking values of genes reference used**

Transcript accumulation of candidate genes was detected in rice roots after infection with *M. incognita*. The stability of each *M. incognita* gene expression during rice infection was analyzed using RefFinder (Xie et al. 2012; <http://www.leonxie.com/referencegene.php>).


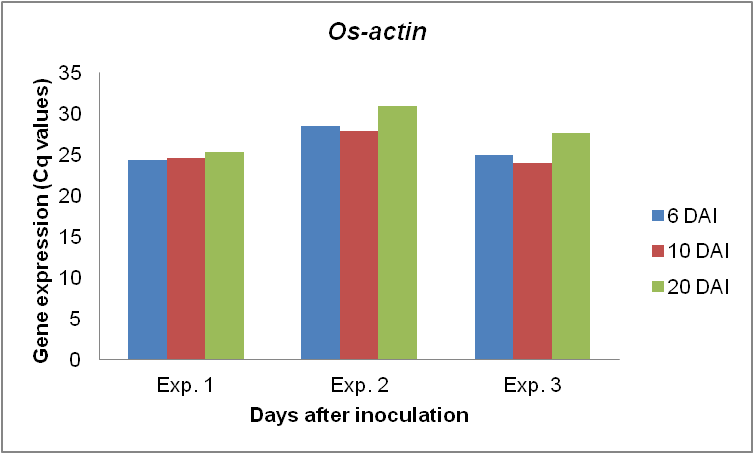


**Figure S2**. **Relative expression of the *Os-actin* gene in *M. incognita*-infested rice *O. sativa* cv. Nipponbare roots.**

Gene expression was measured by reverse transcription-quantitative polymerase chain reaction in plants infested with *M. incognita* at different time points after treatment. Data presented are mean values of two technical replicates. Three independent biological replicates were carried out, with 35 plants per condition (*n=3, each contained 35 plants pooled*).
