# Supplementary figures and images for: Meloidogyne incognita - rice (Oryza sativa) interaction: a new model system to study plant-root-knot nematode interactions in monocotyledons
Source: Rice (N Y). 2014 Sep 22;7:23. doi: 10.1186/s12284-014-0023-4 (PMC4884005; doi:10.1186/s12284-014-0023-4)

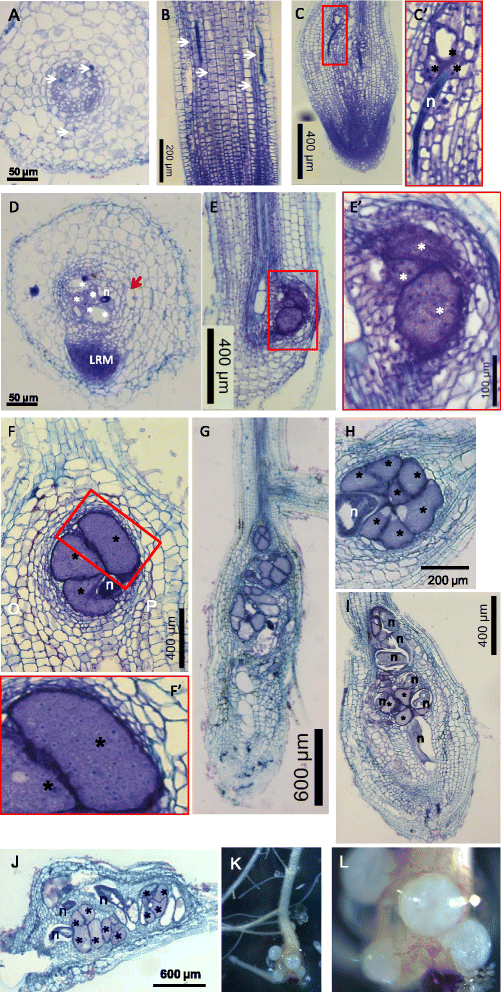

Supplement: Supplementary file 3 — Authors’ original file for figure 1 [file 12284_2014_23_MOESM3_ESM.gif]

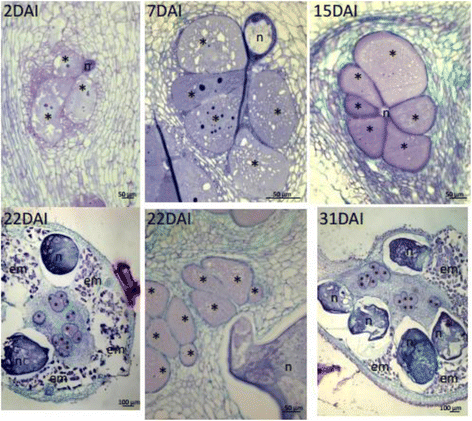

Supplement: Supplementary file 4 — Authors’ original file for figure 2 [file 12284_2014_23_MOESM4_ESM.gif]

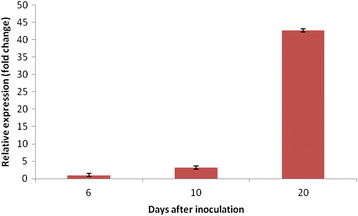

Supplement: Supplementary file 5 — Authors’ original file for figure 3 [file 12284_2014_23_MOESM5_ESM.gif]

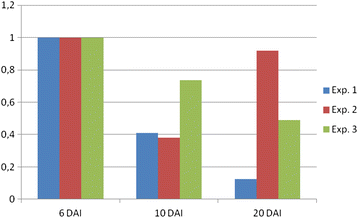

Supplement: Supplementary file 6 — Authors’ original file for figure 4 [file 12284_2014_23_MOESM6_ESM.gif]

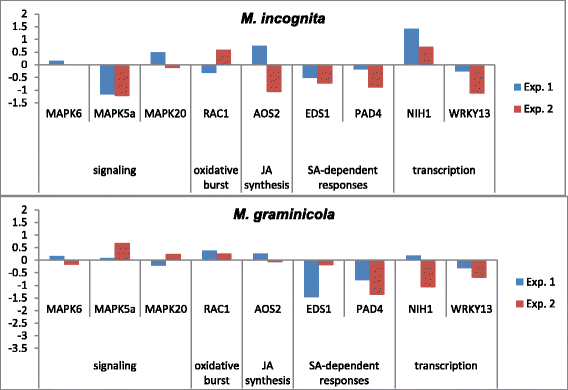

Supplement: Supplementary file 7 — Authors’ original file for figure 5 [file 12284_2014_23_MOESM7_ESM.gif]
